# Concept analysis of health system resilience

**DOI:** 10.1186/s12961-024-01114-w

**Published:** 2024-04-05

**Authors:** Deena Al Asfoor, Celine Tabche, Manal Al-Zadjali, Awad Mataria, Sohel Saikat, Salman Rawaf

**Affiliations:** 1 WHO-EMRO, Cairo, Egypt; 2 WHOCC Imperial College London, London, United Kingdom; 3 MoH-Oman, Muscat, Oman; 4 WHO-HQ, Geneva, Switzerland

**Keywords:** Health planning, Health policy, Delivery of healthcare, Resilient health system, Concept formation, Learning health system

## Abstract

**Background:**

There are several definitions of resilience in health systems, many of which share some characteristics, but no agreed-upon framework is universally accepted. Here, we review the concept of resilience, identifying its definitions, attributes, antecedents and consequences, and present the findings of a concept analysis of health system resilience.

**Methods:**

We follow Schwarz-Barcott and Kim’s hybrid model, which consists of three phases: theoretical, fieldwork and final analysis. We identified the concept definitions, attributes, antecedents and consequences of health system resilience and constructed an evidence-informed framework on the basis of the findings of this review. We searched PubMed, PsycINFO, CINAHL Complete, EBSCOhost-Academic Search and Premier databases and downloaded identified titles and abstracts on Covidence. We screened 3357 titles and removed duplicate and ineligible records; two reviewers then screened each title, and disagreements were resolved by discussion with the third reviewer. From the 130 eligible manuscripts, we identified the definitions, attributes, antecedents and consequences using a pre-defined data extraction form.

**Results:**

Resilience antecedents are decentralization, available funds, investments and resources, staff environment and motivation, integration and networking and finally, diversification of staff. The attributes are the availability of resources and funds, adaptive capacity, transformative capacity, learning and advocacy and progressive leadership. The consequences of health system resilience are improved health system performance, a balanced governance structure, improved expenditure and financial management of health and maintenance of health services that support universal health coverage (UHC) throughout crises.

**Conclusion:**

A resilient health system maintains quality healthcare through times of crisis. During the coronavirus disease 2019 (COVID-19) epidemic, several seemingly robust health systems were strained under the increased demand, and services were disrupted. As such, elements of resilience should be integrated into the functions of a health system to ensure standardized and consistent service quality and delivery. We offer a systematic, evidence-informed method for identifying the attributes of health system resilience, intending to eventually be used to develop a measuring tool to evaluate a country’s health system resilience performance.

**Supplementary Information:**

The online version contains supplementary material available at 10.1186/s12961-024-01114-w.

## Background

On 21 December 2019, the WHO received a report of pneumonia cases with unknown aetiology from China [1]. The new disease, later named coronavirus disease 2019 (COVID-19), spread rapidly to Thailand, Japan and Korea in January 2020 and to most countries across the globe by May 2020 [1]. By 14 January 2024, multiple waves had hit the world, resulting in more than 773.8 million cases and claiming more than 7 million lives [2, 3].

Throughout the pandemic, many governments struggled with managing the ever-increasing numbers of patients with COVID-19 while sustaining essential health services. The difficulty in managing this dual challenge raised questions about our perception of what constitutes a resilient health system, starting with the validity of current health systems frameworks, in particular, those involving monitoring of health security and universal health coverage (UHC), and the ability of those systems irrespective of their maturity to maintain health services during and after a pandemic or a system shock [4].

Resilience, as it relates to health systems, is recognized as an essential prerequisite for UHC and health security. Despite its growing importance, scholarly work in this area rarely went further than definitions and frameworks. In addition, we observed many health systems’ resilience measures in the literature, but there seems to be a gap in justifying the linkages between resilience definitions and how it is measured. A review in 2020 indicated that the recommendations to improve resilience are not informed by theory. As such, there is a need for a practical, evidence-informed, comprehensive framework that can support comparisons between countries, drive plans to progress towards UHC and ensure a systematic approach to tackling emergencies from a health system’s perspective [5].

To fill this knowledge gap, we present a concept analysis for “health system resilience” and propose a conceptual framework that could be used to advance further research and measure resilience to advance health security and UHC efforts.

## Methods

Concept analysis is a means for establishing conceptual clarity about a phenomenon [6]. This inquiry method was extended to healthcare because it is widely thought that any discipline is responsible for building its scientific research base from a set of well-developed concepts for an interest area [7].

Clearly defined concepts are the basic building blocks of theories and knowledge in science and are used to establish a common understanding of a phenomenon across disciplines. Concepts can be poorly defined, understood, unambiguous and undeveloped [8–10].

There are many methods and approaches to concept analysis, and in choosing one, the researcher should be guided by the analysis objectives and concept maturity. The most common yet widely criticized concept analysis method is the Wilson method. This method and others branching from it, otherwise commonly named Wilsonian methods, had been implicated as overly simplistic, lacking scientific rigor and having limited operational use. They also depend on case scenarios, which may not always be applicable or represent the phenomenon discussed. Another commonly used framework is Morse and Rodgers’ evolutionary method. Morse and Rogers propose the idea of ‘concept maturity’ and consider the evaluation of concept maturity a prerequisite for concept analysis [8, 11, 12]. Evaluation of ‘concept maturity’ is a subjective evaluation that considers the clarity of the concept definitions, characteristics, preconditions, outcomes and delineated boundaries.

**Fig. 1:**
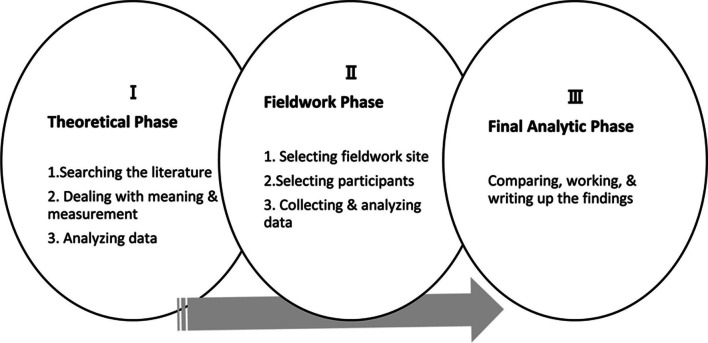
Steps in the Schwartz-Barcott and Kim's hybrid model for concept anlysis

Because of the relatively small number of articles tackling health system resilience, we judged that the health care resilience concept is not mature. Therefore, we chose Schwarz-Barcott and Kim’s hybrid model, which consists of theoretical, fieldwork and final analytical phases (Fig. 1). In this paper, we present the results of the first theoretical phase.

We aim to identify the definitions, attributes, antecedents and consequences of health system resilience and better understand it from the literature point of view. After this, we propose a framework of the domains that could be used to measure health system resilience. The measure of health system resilience will not be explored in this paper.

The theoretical phase of Schwarz-Barcott and Kim’s hybrid model consists of three elements: searching the literature, dealing with meaning and measures and analysing the data.

### Searching the literature

**Fig. 2:**
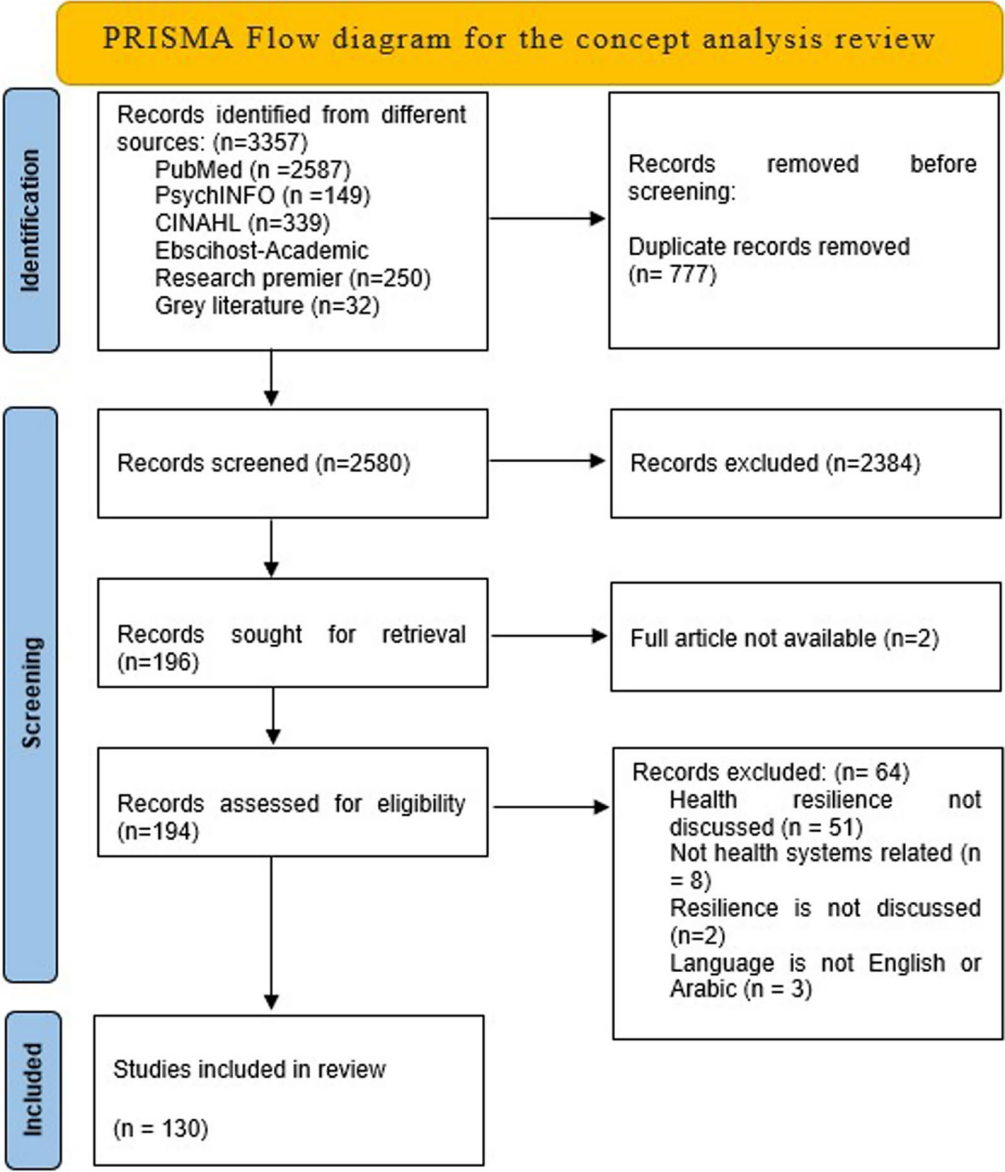
Prisma flow chart for the concept analysis of health systems resilience

A PubMed search of the term “Health System Resilience” on 16 June 2021, yielded 4704 entries, and most of the articles were published in the past 10 years (Fig. 2).

For health system resilience (HSR), a preliminary PubMed search on 29 June 2020, using the keywords “resilience health system” and “resilient health system”, showed 3510 and 1772 entries, respectively, with the oldest publication being from 1977 [13].

*Data sources and inclusion criteria*: To develop the most effective search strategy, we explored several search strategies to understand the impact of alternative definitions on the number of articles identified. A search for MeSH terms “resilience” and “resilient” combined with “health system” was found to yield the highest number of records; therefore, we used this as a search strategy for all the databases used.

To improve the search strategy coverage, we applied the search on PubMed and EMBASE. As the topic is also relevant to public health, it was important to include the Cumulative Index for Nursing and Allied Health Services (CINAHL) [14–16]. We conducted the last search on 1 July 2021, and created an alert on PubMed to capture additional articles until 30 December 2021. We also searched the WHO websites and contacted the organization’s various departments for additional literature.

*Inclusion criteria*: We included all observational, case–control, randomized controlled trials or review studies that measured resilience as a dependent or independent variable in all healthcare system settings with no exclusion on participants or interventions basis.

*Exclusion criteria*: Studies or papers that did not discuss resilience or examine the concept from a health system perspective were excluded. Examples of excluded articles are those focussing on the patient, personal resilience, etc.

### Data analysis

Data extraction and coding: Three researchers (DA, MZ and CT) reviewed the abstracts following the Cochrane guidelines for systematic reviews. We eliminated all duplicates and downloaded 2580 titles and abstracts on the Covidence program for systematic reviews management [17]. Two reviewers screened each abstract using the Covidence program, and disagreements were resolved by discussion with the third reviewer. Full-text articles of eligible titles were retrieved and screened for eligibility using the same methodology [18].

After agreeing on the manuscripts, the three reviewers used a data extraction form to examine the publications for definitions, attributes, antecedents and consequences (Additional file 1: Annex 1). Attributes are the components or characteristics of a concept. Antecedents are events or phenomena leading to resilience. Consequences or the results of the concept are the events following the concept realization. A list of attributes, antecedents and consequences from the three researchers were compared, and a unified list was produced through consensus [8]. Simultaneously, we screened the articles reviewed for definitions, frameworks and tools.

### Dealing with meaning and measures

The initial search yielded 2648 articles, of which 68 duplicates were removed, leaving 2580 abstracts for screening. We found 2348 titles ineligible to be included. Among the 196 eligible papers, the authors of 51 articles did not explain the relation of resilience to the work presented in each paper, 8 discussed resilience but not in a health systems context, 3 were in languages other than English (French and Farsi), 2 did not discuss resilience and we could not access two full articles. Finally, we included 130 studies in the analysis.

The word resilience originates from the Latin prefix ‘re-’ (back) and the verb ‘salire’ (to jump, leap). In science, it has long been used by engineering and material science to describe the ability of a material to absorb energy without losing its original form or characteristics [19, 20]*.*

In psychology, resilience is the individual capacity to cope with crises, losses or hardships without negative consequences. It is the ability to absorb and adapt to a changing environment. It is also defined as the ability of the system to withstand a significant disruption within acceptable degradation parameters and to recover within an acceptable time and composite costs and risks [21, 22].

The Cambridge Dictionary defines resilience as the ability of a substance to return to its usual shape after being bent, stretched or pressed [19]. In a health system context, this could be interpreted as the ability of the system to adapt. As such, health system resilience (HSR) can be defined as the capacity of health systems to absorb, adapt and transform when exposed to a shock such as a pandemic, natural disaster or armed conflict. A resilient health system maintains core functions and structure when a crisis hits. In addition, this system learns from lessons learned through the crisis and reorganizes “symptoms” of an approaching crisis [5, 23].

Several surrogates for resilience are being used; some view resilience as a dynamic behavior that reflects the system’s malleability. For example, preparedness, responsiveness, adaptability and adjustment to stress are surrogates that reflect the dynamic nature of resilience [4, 5, 23–27]. Other surrogates imply that resilience is a feature of the system, such as strengthening, transformative, adjusting to stress, coping strategies, sustainability, absorptive capacity, surge capacity and so on [4, 5, 25, 26, 28–31].

## Results

### Antecedents

Antecedents in this study were the prerequisites for resilience, and these can also be health system prerequisites such as system enablers or other external factors that boost resilience. We decided to extract them from the papers so that each antecedent should be in one sentence, and there should be no more than five.

1. *Decentralization*: Governments at different levels, community health committees and health boards have considered decentralization the main prerequisite for HSR [32–35]. Decentralization, the opposite of centralization, is defined as the transfer of formal responsibility and power to make management, distribution (of medicines) or financing decisions of health services to organizationally separate actors. This approach promotes responsiveness to communities and transforming disease patterns, moving away from central government and other delays [34, 36]. Decentralization in this context groups many other antecedents of HSR, for example, medicines supply, governance, policies and leadership that monitor, evaluate, and strengthen the system [34, 37–43].
2. *Funds, investment and resources*: A long-term plan for funding and resources should be in place to ensure a sustainable and efficient health system [32, 37–39, 42–49]. The resources should cover infrastructure, service delivery, products, technologies, protection equipment, temporary shelter, transportation and food.
3. *Staff environment and motivation*: These were pointed out in many studies as a pillar of HSR. The workforce should be able to function through any situation, be trained well, and be kept satisfied [33–35, 37, 41, 49–52]. To that end, motivational interests should be aligned, the number of conflicts within health teams should be kept to a minimum and dealt with promptly and threats should be carefully managed. The health workforce also requires constant training and attention to their wellbeing and health [23, 34, 41, 44, 52].
4. *Integration and networking*: These bring together diverse actors and sectors, establishing a local, regional and national framework for rapid information sharing, decision-making and action [5, 27, 29, 31, 33, 34, 37, 39, 42, 47, 49, 53–58].
5. Diversification of the staff from different fields, backgrounds and education has been mentioned, allowing more than one perspective to be considered when addressing a specific problem [44, 59]. Public Health can be found in every profession, and staff members from all backgrounds should be competent enough to tackle emergencies. Awareness/social learning/community engagement was discussed to make sure that the health staff could be able to teach the public and raise awareness in every setting [23, 28, 29, 33, 34, 41, 42, 60].

### Attributes

Attributes are identified as the components or characteristics of a concept. We identified several attributes of the health system resilience (HSR). Resilient health systems are thought to have the following attributes:

1. Availability and flexibility of resources and funds: These include multiple sources of funding, equipment, medical products and technologies, commitment to long-term investment in health services and the ability to reallocate resources when needed and ensure adequate human resources that are accountable and committed [23, 29, 31, 37, 45, 57, 58, 60–69].
2. Adaptive capacity: For a health system to be resilient, it should be able to adapt and move responsibilities and authorities horizontally and vertically: horizontally means across the health system components and vertically along the health system levels. Being adaptive means that a resilient health system should allow for “backup” and have “shock-absorbers” capacity. Planning for post-event recovery should be integrated into the system’s architecture [4, 23, 25, 27, 29–32, 41, 53, 58, 60, 61, 70–72].
3. Transformative capacity: Resilient health systems have a transformative capacity that enables them to reorganize and adapt to change while maintaining original functions and ensuring long-term sustainability through self-regulation and the ability to reshape how care is delivered. Such health systems can always ensure the continuity of health services [4, 27, 29, 30, 37, 59, 61].
4. Learning and advocacy: Resilient health system communicates information for awareness, knowledge exchange or learning, including appropriate disease surveillance systems, and they learn from experiencing aftershock, stress, pressure or uncertainties [4, 5, 23, 25, 27, 38, 41, 57, 64, 70, 72–77].
5. Progressive leadership: The leadership and management of resilient health systems are thought to have several unique characteristics, such as having inclusive decision-making, engaging the community with the health system and building social networks, partnerships and trusting relationships. Resilient health systems encourage innovation, provide a creative climate and believe in diversity. They provide continuous training and development opportunities and implement adaptive staffing adjustments when required. These systems are flexible, responsive and proactive and work on strengthening their infrastructure [23, 29, 41, 59, 78, 79].

### Consequences

As mentioned, consequences are the expected results of a resilient health system. This impact could be on the health system or the population affected. In the review, four significant resilience consequences emerged, and these are:

1. Improved health system performance: Health quality and human resources deployment have been enhanced because of resiliency in the health system in Yobe [33]. In Lebanon, decentralization and delegation to lower levels were witnessed as the system responded to the influx of migrants [80]. Improved capacity to absorb the increased demand for health services and improved human resources management are necessary outcomes of a resilient health system [41].
2. Balanced governance: It is argued that a resilient system shifts political imagination and values. Additionally, a solid governance structure can be challenged by resilience. Therefore, a balance should be struck between legitimacy and deliberation in policy and practice to reach a well-governed and sustainable health system [56].
3. Improved expenditure on health and financial management: While responding to crises, health systems require increased and more efficient spending [37, 71]. In the face of crisis, while there is a time-bound demand for increased investment for surge capacity, a transformative health system could change policies and practices to support integration and smart investment and improve allocative efficiency and fiscal governance.
4. Maintaining essential health services and supporting universal health coverage and health security: The shock to the health system is likely to disrupt essential health services and expose the faults and health system gaps as seen in the context of COVID-19, but this can also create a higher level of awareness and motivate policymakers to adopt health system policies (both emergency specific and routine), increase the reliance on primary care and improve fair and adequate health coverage to all. The policy change and improvement in the health system, in turn, will reduce excess mortality and morbidity, human suffering and the socio-economic cost associated with the crisis [49, 52, 70, 81].

## Discussion

### Frameworks and methods of measurement

A review of the methods and frameworks revealed several frameworks used to measure resilience. Some frameworks did not mention resilience, making it challenging to understand the rationale behind using them to measure HSR, such as the WHO’s Framework for Action and the framework for strengthening health emergency preparedness in cities and urban settings [82, 83]. The WHO emergency preparedness framework and the SmartResilience approach focussed on emergency response utilizing a risk management approach, and the health system components were not addressed sufficiently [84, 85]. A few frameworks focussed on one or more components of the health system, such as Gilson et al.’s everyday HSR framework that focussed on primary healthcare and health workforce functioning [40], Bruneau et al.’s seismic resilience of communities [86], Kruse et al.’s emBRACE framework [87] and Robertson et al.’s model of personal resilience [88]. The resilience framework for public health emergency preparedness [23], the resilient health system framework and the resilient index were the most comprehensive and relevant frameworks. However, linking indicators to each of the framework elements remains a challenge [89, 90] (Additional file 1: Annex 4)*.*

All of the above frameworks have the following concepts in common:

- Leadership and management capacities: These include knowledge, interdependence and legitimacy [4, 43, 91], in addition to cognitive, conceptual and behavioral capacities and being absorptive, adaptive and transformative [43]. Further, these capacities also include the ability to take action [87], confidence, purposefulness [87], collaborative networks, community engagement and availability of planning processes [25].
- Resources: These include different types of resources such as general resources and capacities [90], human resources [4, 24, 27], financial resources [4, 27], medical products and services as well as social support [4, 49].
- Learning and evaluation systems: These include formal or informal learning to prevent hazards [87, 90], information and information management systems and risk analysis and surveillance and monitoring [4, 24, 27]*.*

Outbreaks and manmade crises create unpredictable emergencies, so the health systems possess sufficient dynamicity to respond effectively. Some countries responded more efficiently to the COVID-19 pandemic than others [92]. For example, the Irish government established a dedicated reform office in Ireland that oversaw policy change, budget expansion and innovation. COVID-19 diagnostics and treatment were free, and telemedicine practice was adopted, among other interventions. An increased budget supported this; nevertheless, the challenge remains as to whether these transformations will continue after the pandemic [93].

Dynamicity is the shared component among all attributes of resilience. The WHO approach to health systems proposes the hardware building blocks but neglects that these same pillars should be flexible to withstand chronic and acute crises [94, 95].

During the pandemic, countries altered their service delivery models of care and adopted innovative strategies to deliver care and medicines. Improved budgeting and purchasing mechanisms were implemented to ensure the timely availability of products and medicines. Had the structures been stagnant, no matter how robust, the health systems would have collapsed, as the case was in several high-performing health systems [96].

**Fig. 3:**
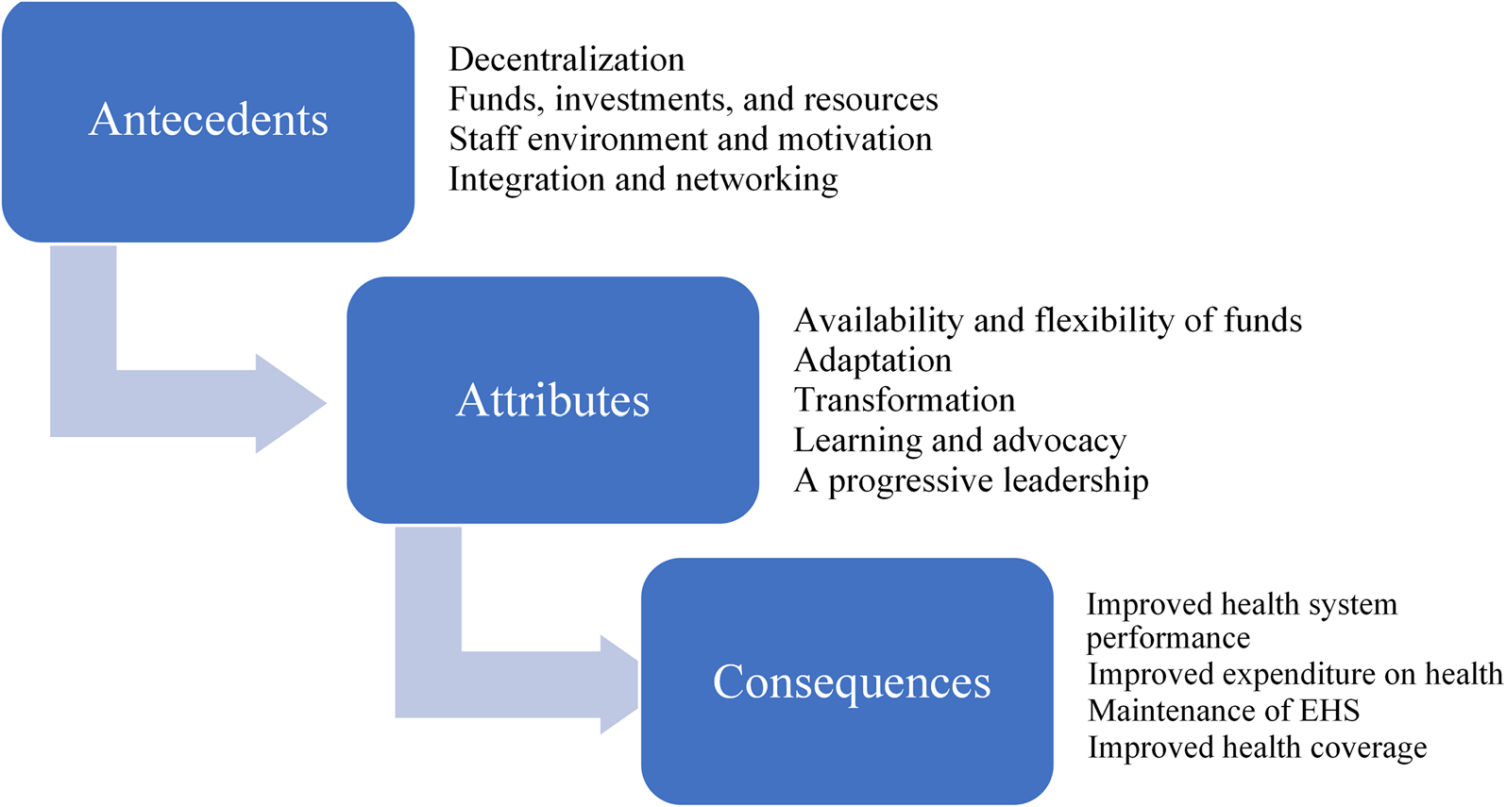
Framework of health system resilience

It is important to be able to develop resilience indicators that are based on an evidence-based framework. A framework for measuring HSR should be standardized worldwide. A unique language of resilience in health in emergency situations can be derived from the work done in this study. The main points to consider are sufficient resources in the health system for the foundation (workforce, infrastructure, technology and utility) and medical products, which should have a particular system, keeping them in order all year round and not only during emergencies. The second point is competent staff who could lead and manage the organization in every department, especially epidemiology and public health analysis. All staff require regular competency-based training in public health emergencies. The third point is to make a system for monitoring and evaluation that keeps data collection, analysis, maintenance and sanitation in check. The cross-cutting requirement is power and flexibility in public health governance to ensure multi-sectorial coordination and mobilization of resources as and when needed in the face of fast-evolving emergencies (Fig. 3).

### Strengths and limitations

This review was conducted using robust scientific review methods. Each record was reviewed by more than one researcher, sometimes three, to ensure consistent views and not to miss any critical elements. We focussed on ensuring an evidence-based and comprehensive yet practical approach to identifying the resilience framework elements. This is the first concept analysis of health system resilience, which can be used to enhance health systems evaluations and reports.

Our review has some limitations; for example, papers published on health system resilience did not discuss the tools used to measure health system resilience; most were opinion pieces on what health system resilience should include or look like. Some of the selected papers were unavailable, or they were not included because of language limitations. Since the onset of the COVID-19 pandemic, hundreds of papers have been published about health systems and included the word ‘resilience’ or ‘resilient’, but only discussed the staff and not the entire health system, which was difficult to separate due to bias. The scope and quality of papers differed widely depending on the region the paper came from (Fig. 2).

## Conclusion

This review widens the scope of health system resilience to ensure reliability and repeatability at the global level despite the difference in resources and priorities. It compiled all the papers published and accessible on health system resilience until the end of 2021. It summarizes the main components repeated in most studies to help us reach the primary indicators that will help develop a standardized health system resilience measurement tool with definitions, antecedents, attributes and a framework.

In this paper, we have established the domains and attributes as a first step in developing a set of indicators for health system resilience. The framework presented can be used to select resilience indicators through a systematic and evidence-informed process.

### Supplementary Information

**Additional file 1: Annex 1.** Data extraction form. **Annex 2.** Some definitions of resilience. **Annex 3.** Surrogates of resilience in the context of health system. **Annex 4.** Frameworks used in measuring health system resilience (HSR).

## Data Availability

Any additional data or materials can be provided upon request from the corresponding author.

## Abbreviations

COVID: Coronavirus disease
EHSR: Everyday health system resilience
EVD: Ebola virus disease
HSR: Health system resilience
RI: Resilience index
SI: Stress index
UHC: Universal health coverage
WHO: World Health Organization
