# Supplementary material for: Concept analysis of health system resilience
Source: Health Res Policy Syst. 2024 Apr 5;22:43. doi: 10.1186/s12961-024-01114-w (PMC10996206; doi:10.1186/s12961-024-01114-w)
Supplement: Supplementary file 1 — Additional file 1: Annex 1. Data extraction form. Annex 2. Some definitions of resilience. Annex 3. Surrogates of resilience in the context of health system. Annex 4. Frameworks used in measuring health system resilience (HSR). [file 12961_2024_1114_MOESM1_ESM.docx]

Annex 1: Data extraction form

| **S.N.** | **Author (date)** | **Type of Study** | **Setting** | **Surrogate terms** | **Attributes** | **Antecedents** | **Consequences/ outcomes** | **Remarks** |
| --- | --- | --- | --- | --- | --- | --- | --- | --- |
|  |  |  |  |  |  |  |  |  |
|  |  |  |  |  |  |  |  |  |
|  |  |  |  |  |  |  |  |  |
|  |  |  |  |  |  |  |  |  |

Annex 2: Some definitions of resilience

| **Definition** | **Source** |
| --- | --- |
| The degree of change a system can undergo while maintaining its functionality. | (5) |
| The capacity of a system to experience shock without major consequences, determined by the adaptability and robustness of a system to potential acute shocks or chronic stress | (33) |
| The ability… to manage change, by maintaining or transforming…standards in the face of shocks or stresses.... without compromising… long-term prospects | (64) |
| The capacity that enables the health system to return to ‘normal and usual’ function when faced with a shock/stressor | (66) |
| “Resilience (…) can be defined as the capacity of a dynamic system to withstand or recover from significant challenges that threaten its stability, viability, or development | (66) |
| Resilience is the capacity of a health system to absorb internal and external shocks, and maintain functional health institutions while sustaining achievements | (38) |
| The capacity of health systems to continue to deliver good quality services in the face of these chronic challenges is what we have elsewhere called everyday resilience | (40) |
| A system’s ability to continue to perform and meet its objectives in the face of challenges | (45) |
| A system’s capacity to absorb disturbances, while learning from them and reorganising | (45) |
| The capacity of health actors, institutions, and populations to prepare for and effectively respond to crises; to maintain core functions when a crisis hits; and, informed by lessons learned during the crisis, to reorganise if conditions require it’ | (96) |
| Ability to absorb changes of variables and maintain relationships between different populations | (67) |
| A measure of the amount of change a system can experience while maintaining the same controls on structure and function’ | (68) |
| Capability of a health system to mitigate the impact of major external disruptions on its ability to meet the needs of the population during the disaster’ | (69) |
| Community resilience proposes that the key to a good disaster response lies in communities, and their ability to ‘prepare, respond, and recover’ from major events through a range of measures including increased social connectedness, adaptive health and social systems and emergency preparedness planning | (70) |
| Take an ‘everyday resilience’ perspective to understand the ability of health workers in dealing with everyday challenges | (41) |
| The ability of the health system (a clinic, a ward, a hospital, a country) to adjust its functioning prior to, during, or following events (changes, disturbances, and opportunities), and thereby sustain required operations under both expected and unexpected conditions | (72) |
| The ability of any social system to proactively adapt to disturbances | (73) |
| The abilities of different groups (families, populations, communities, health workforce, institutions, organizations, individuals, systems society) to react in the face of change, to anticipate, to withstand, and to bounce back | (27) |
| Health systems resilience emphasises the capacity of health systems to maintain their essential functions during to crisis – sudden or expected, internal or external – in order to absorb them; | (27) |
| The resilience thinking associated to health systems study highlights the abilities of different entities to adapt and transform to face destabilising experiences or shocks. | (27) |
| Capacity of the health system to provide quality services in the face of chronic challenges such as structural and policy instability, unpredictable staff, changing patient and community expectations, etc. | (40) |
| Resilient health system is capable to anticipate, respond to, cope with, recover from and adapt to climate-related shocks and stress, so as to bring sustained improvements in population health, despite an unstable climate | (74) |
| The capacity of health actors, institutions, and populations to prepare for and effectively respond to crises; maintain core functions when a crisis hits; and, informed by lessons learned during the crisis, reorganise if conditions require it | (30) |
| The capacity to recover quickly from a shock or, in reference to materials, the ability of an object to bounce back into shape (elasticity) | (75) |
| The health system’s ability to prepare, manage (absorb, adapt and transform) and learn from shocks, | (75) |
| A system’s “ability to absorb change and disturbance and still maintain the same relationships between populations or state variables | (67) |
| The capacity of an individual, population or system to absorb a shock, while still retaining the fundamental functions or characteristics of the original state | (76, 77) |
| The capacity to absorb internal and external shocks and maintain functional health institutions while sustaining achievements | (38) |
| Its capacity to adapt, absorb and transform when exposed to a shock such as a pandemic, natural disaster, armed conflict or a financial crisis and still retain the same control over its structure and functions | (78) |
| Able to adapt effectively to changing environments and tackling significant challenges with limited resources | (79) |
| Able to adapt its functioning to absorb a shock and transform if necessary to recover from disaster | (28) |
| The capacity of health actors, institutions, and populations to prepare for and effectively respond to crises, maintain core functions when a crises hits, and informed by lessons learned during the crisis, reorganise if conditions require it | (91) |
| Can anticipate, respond to, cope with, recover from and adapt to climate-related shocks and stress to provide sustained improvements in population health despite an unstable climate | (97) |
| The ability to withstand shocks, be they natural or man-made disturbances | (4) |
| The ability to manage and cope with threats, challenges and emergencies while maintaining the normal functions and services of a health system; a system can also learn from these experiences and develop and evolve its functionality to become even stronger | (4) |
| Its capacity to adapt to meet the healthcare needs of the population which requires dependable and adaptable resources including supply chains, reliable funding for healthcare, and reserve healthcare worker capacity (loss of healthcare workers due COVID-19 infection or fear of acquiring the infection should be expected during the epidemic | (82) |
| The maintenance of positive adjustment under challenging conditions such that the organisation emerges from those conditions strengthened and more resourceful’ | (98) |
| the ability of the individual to adapt to adverse conditions, trauma, or stress | (99) |
| The system being able to adapt its functioning to absorb a shock and transform, if necessary, to recover from disaster | (93) |
| As institutions’ and health actors’ capacities to prepare for, recover from and absorb shocks, while maintaining core functions and serving the ongoing and acute care needs of their communities6 | (91) |
| The ability to continue service delivery in the face of extraordinary shock | (95) |
| the ability of health systems to maintain service provision in times of chaos and uncertainty | (100) |
| System’s capacity to respond adaptively to an external shock that challenges its functioning. | (95) |
| The ability to anticipate, prepare for, and adapt to changing conditions and withstand, respond to, and recover rapidly from disruption | (101) |
| The ability to understand risks, anticipate, prepare for, and adapt to changing conditions and withstand, respond to, and recover rapidly from disruption | (102) |
| The ability to understand and anticipate the risks – including new/emerging risks threatening the critical functionality of the infrastructure, prepare for anticipated or unexpected disruptive events, optimally absorb/ withstand their impacts, respond and recover from them, and adapt/transform the infrastructure or its operation based on lessons learned, thus reducing the critical infrastructure fragility | (103) |
| Everyday resilience is ‘the maintenance of positive adjustment under challenging conditions such that the organisation emerges from those conditions strengthened and more resourceful’ | (104) |
| Resilient systems are those that rapidly acquire information about their environments, quickly adapt their behaviours and structures to changing circumstances, communicate easily and thoroughly with others, and broadly mobilise networks of expertise an | (105) |
| The ability of the health system to respond quickly during a health emergency | (92) |
| A resilient health system is one able to absorb stresses and shocks, adapt to changes and transform if needed | (29) |
| Disaster resilience: resilience is defined as the ability to minimise the costs of a disaster, to return to a state as good as or better than the status quo ante, and to do so in the shortest feasible time | (106) |
| Community resilience: the ability of social units ... to mitigate hazards, contain the effects of disasters when they occur, and carry out recovery activities in ways that minimise social disruptions and mitigate the effects of future (disasters | (89) |
| A multi-dimensional construct, resilience is defined as the capacity of individuals, families, communities, systems, and institutions to anticipate, withstand and/or judiciously engage with catastrophic events and/or experiences; actively making meaning with the goal of maintaining normal function without fundamental loss of identity | (107) |
| Is the intrinsic capacity of a system, community or society predisposed to a shock or stress to adapt and survive by changing its non-essential attributes and rebuilding itself | (108) |
| The capacity of a system, community or society potentially exposed to hazards to adapt, by resisting or changing, in order to reach and maintain an acceptable level of functioning and structure | (90) |
| The degree to which the social system is capable of organising itself to increase this capacity for learning from past disasters for better future protection and to improve risk reduction measures | (67) |
| The ability of a system and its component parts to anticipate, absorb, accommodate, or recover from the effects of a potentially hazardous event in a timely and efficient manner, including through ensuring the preservation, restoration, or improvement of its essential basic structures and functions | (109) |
| The capability of an entity - “community, an organisation, or a natural system - to prepare for disruptions, to recover from shocks and stresses, and to adapt and grow from a disruptive experience | (110) |
| The ability of households, communities and nations to absorb and recover from shocks (shocks reflect both infrequent catastrophic events that characterise intensive risk, and frequent low-impact events that characterise extensive risk and which may cumulatively have great impacts over time), whilst positively adapting and transforming their structures and means for living in the face of long-term stresses, change and uncertainty | (111) |
| A system that is capable to anticipate, respond to, cope with, recover from and adapt to climate related shocks and stress, so as to bring sustained improvements in population health, despite an unstable climate. | (97) |
| The ability of individuals, families, communities, systems, and institutions to anticipate, withstand and/or judiciously engage with catastrophic events and/or experiences. | (98) |
| The capacity of health actors, institutions, and populations to prepare for and effectively respond to crises; maintain core functions when a crisis hits; and, informed by lessons learned during the crisis. | (91) |
| The capacity of a health system to both survive sudden shocks, such as disease outbreaks, and the ongoing strain of structural, policy, managerial and community instability | (104) |
| The ability of a health system to continue providing normal services in response to a crisis. | (112) |
| The capacity/intrinsic ability of a social system (e.g., an organisation, city or society) to proactively adapt to and recover from disturbances that are perceived within the system to fall outside the range of normal and expected disturbances/conditions so that it can sustain required operations. | (42) |
| The capability of the public health and healthcare systems, communities, and individuals to prevent, protect against, quickly respond to and recover from health emergencies, particularly those whose scale, timing or unpredictability threatens to overwhelm routine capabilities. | (52) |
| The capacity of a [health system] to absorb, adapt [or] transform when exposed to a shock such ... armed conﬂict and still retain the same control over its structure and functions | (78) |
| Is the capability of a health system to prepare, respond and reorganise under conditions of stress, is posited to protect the population from excess morbidity and mortality | (113) |
| Is the capacity to prepare for disruptions, to recover from shocks and stresses, and to adapt and grow from a disruptive experience. Resilience is a process; there can be different levels of resilience. Resilience incorporates the concept of emergency preparedness. For an overall health system to be sustainable, to maintain basic functioning in times of shock, it must have high resilience. | (25) |
| The capacity of a system to absorb change but continue to retain essentially the same identity and function | (114) |
| The capacity to sustain essential services provision, even in the context of shock events | (45) |
| The ability to recover from or adjust to shocks | (78) |
| Is the ability of the health care system (a clinic, a ward, a hospital, a country) to adjust its functioning prior to, during, or following events (changes, disturbances, and opportunities), and thereby sustain required operations under both expected and unexpected conditions | (115) |
| The capacity of a system to absorb disturbance and reorganise while undergoing change so as to still retain essentially the same function, structure, identity, and feedbacks. | (115) |
| A system’s capacity to absorb, adapt, anticipate and transform when exposed to external threats— or forecast shocks that bring about new challenges and opportunities—and still retain control over its remit and pursuit of its primary objectives and functions.1 | (116) |
| The capacity of health actors, institutions, and populations to prepare for and effectively respond to crises; maintain core functions when a crisis hits; and, informed by lessons learned during the crisis, reorganise if conditions require it. | (32) |
| The ability of a health system to meet the sudden or extended increase in demand for medical care in the event of a crisis | (32) |
| The adaptability of a system to emerging needs and instability | (30) |
| The ability of the system or process to deal with the shock or stress is based on the levels of exposure, the levels of sensitivity and adaptive capacities. | (34) |
| The ability of populations to prepare for and effectively respond to crises, by reorganising systems to manage the new conditions while maintaining core functions. | (4) |
| The capacity to prepare for and effectively respond to crises while maintaining core health system functions pre-, during, and post-crisis. | (27) |
| The ability of the health systems and their actors to prepare for and adapt to climate-related shocks. | (117) |
| The capacity of social, economic, and environmental systems to cope with a hazardous event or trend or disturbance, responding or reorganising in ways that maintain their essential function, identity, and structure, while also maintaining the capacity for adaptation, learning, and transformation. | (109) |
| The resilience of a health system is its capacity to respond, adapt, and strengthen when exposed to a shock such as a disease outbreak, natural disaster or conflict. | (100) |
| The ability of an organisation to anticipate, prepare for, and respond and adapt to incremental change and sudden disruptions in order to survive and prosper | (118) |

Annex 3: Surrogates of resilience in the context of health system

| Reference | Term used |
| --- | --- |
| Barassa, 2018 (45) | Bounce back |
| Biddle 2020 (5)  Turenne 2019 (27)  Fridell 2020 (4)  Ling 2017 (46)  Brooke-summer 2019 (43)  Labarda 2017 (44) | Strengthening |
| Biddle 2020 (5)  Brown 2018 (119)  Turenne 2019 (27)  Fukuma 2017(35)  Hanefeld 2018 (28)  Chamberland- Rowe 2019 (32) | Responsiveness |
| Brown 2018 (119)  Turenne 2019 (27)  Fridell 2020 (4)  Hanefeld 2018 (28)  Kagwanja 2020 (31)  Long 2019 (120)  Chamberland-Rowe 2019 (32) | Adaptability |
| Brown 2018 (119) | Adjustment to stress |
| Turenne 2019 (27)  Witter 2017 (36)  Koeva 2020 (29) | Coping strategies |
| Turenne 2019 (27) | Sustainability |
| Turenne 2019 (27)  Fridell 2020 (4)  Kagwanja 2020 (31) | Transformation |
| Ebi 2016 (30)  Fridell 2020 (4)  Kagwanja 2020 (31) | Absorptive capacity Absorption |
| Fridell 2020 (4) | Maintenance |
| Hunte 2020 (121) | “build back better”. |
| Khan 2018 (24)  Rogers 2017 (25)  Labarda 2017 (44) | Preparedness |

Annex 4: Frameworks used in measuring health system resilience (HSR)

| Reference | Framework name | Results | Comments and limitations |
| --- | --- | --- | --- |
| 1. Robertson IT, Cooper CL, Sarkar M, Curran T. Resilience training in the workplace from 2003 to 2014: A systematic review. Journal of Occupational and Organizational Psychology. 2015;88(3):533-62. (82) | Robertson Cooper Model of Personal Resilience | provide a systematic review of work-based resilience training interventions | Evaluates work-based resilience training and is not relevant to health systems resilience |
| 1. World Health O. Everybody's business -- strengthening health systems to improve health outcomes : WHO's framework for action. Geneva: World Health Organization; 2007. (83) | WHO Framework for Action | WHO health systems framework | Does not discuss resilience |
| 1. Khan Y, O'Sullivan T, Brown A, Tracey S, Gibson J, Genereux M, et al. Public health emergency preparedness: a framework to promote resilience. Bmc Public Health. 2018;18. | Resilience framework for public health emergency preparedness | Describe the essential elements of a resilient public health system and how the elements interact as a complex adaptive system. | This is a very informative framework for achieving resilience. It is based on focus groups discussions in Canada. The framework is complex, and although it has many elements, it difficult to operationalize. |
| 1. World Health Organization. A strategic framework for emergency preparedness. Geneva: World Health Organization; 2016. | WHO Emergency Preparedness Framework Resilience | Governance   - National policies and legislation that integrate emergency preparedness. - Plans for emergency preparedness, response and recovery - Coordination mechanisms   Capacities   - Assessments of risks and capacities to determine priorities for emergency preparedness - Surveillance and early warning, information management - Access to diagnostic services during emergencies - Basic and safe health and emergency services - Risk communications - Research development and evaluations to inform and accelerate emergency preparedness.   Resources   - Financial resources for emergency preparedness and contingency funding for response - Logistics mechanisms and essential supplies for health - Dedicated, trained and equipped human resources for emergencies. | Comprehensive, focuses on risk assessment and response during emergencies, but not the health system components. |
| 1. World Health Organization. Framework for strengthening health emergency preparedness in cities and urban settings. Geneva: World Health Organization; 2021. Contract No.: CC BY-NC-SA 3.0 IGO. | Framework for strengthening health emergency preparedness in cities and urban settings | The elements discussed  - Governance and financing for health emergency preparedness  - Multisectoral coordination for preparedness  - High population density and movement  - Community engagement and risk and crisis communication  - Groups at risk of vulnerability  -Data, evidence and information  - Commerce, industry and business  - Organisation and delivery of health and other essential services | Resilience had a small portion of the discussion, and the relationship between preparedness and resilience is not clarified. |
| 1. Jovanović A, Klimek P, Renn O, Schneider R, Øien K, Brown J, et al. Assessing resilience of healthcare infrastructure exposed to COVID-19: emerging risks, resilience indicators, interdependencies and international standards. Environ Syst Decis. 2020;40(2):252-86. | SmartResilience | The approach suggested is composed of a matrix triangulating four phases: (understand risk, anticipate and prepare, absorb/withstand, and respond/recover) and four dimensions: 1. System/physical, 2. Information, data, 3. Organizational/ business, 4. Societal/ political and 5. Cognitive, decision making | This framework focuses on the response to emergencies and does not tackle it from the health system design and functioning perspective. |
| 1. Kruse S, Abeling T, Deeming H, Fordham M, Forrester J, Jülich S, et al. Conceptualizing community resilience to natural hazards – the emBRACE framework. Nat Hazards Earth Syst Sci. 2017;17(12):2321-33. | emBRACE Framework | The framework is composed of three components: resources and capacities, actions, and learning | The focus of this framework is on the community resilience. |
| 1. Bruneau M, Chang SE, Eguchi RT, Lee GC, O'Rourke TD, Reinhorn AM, et al. A Framework to Quantitatively Assess and Enhance the Seismic Resilience of Communities. Earthquake Spectra. 2003;19(4):733-52. | Seismic resilience of communities |  | The focus is on community resilience |
| 1. United Nations. Hyogo framework for action 2005-2015: Building the resilience of nations and communities to disasters: Extract from the final report of the World Conference on Disaster Reduction. Kobe- Japan2005. | Hyogo framework for building the resilience of nations and communities to disasters | The priorities for action for improving resilience of nations and communities to disasters are:   - Ensure that disaster risk reduction is a national and a local priority with a strong institutional basis for implementation - Identify, assess and monitor disaster risks and enhance early warning - Use knowledge, innovation and education to build a culture of safety and resilience at all levels - Reduce the underlying risk factors - Strengthen disaster preparedness for effective response at all levels 20 | This is a policy document and does not tackle elements of the health system. |
| 1. Gilson L, Ellokor S, Lehmann U, Brady L. Organizational change, and everyday health system resilience: Lessons from Cape Town, South Africa. Soc Sci Med. 2020;266:113407-. | Everyday health system resilience framework | Considers resilience to be adaptive, absorptive, and transformative. | Tackles health system focusing on primary healthcare, and elaborates on the health workforce functions. |
| 1. Kruk ME, Myers M, Varpilah ST, Dahn BT. What is a resilient health system? Lessons from Ebola. The Lancet. 2015;385(9980):1910-2. 2. Kruk ME, Ling EJ, Bitton A, Cammett M, Cavanaugh K, Chopra M, et al. Building resilient health systems: a proposal for a resilience index. BMJ. 2017;357:j2323. | Resilient health system framework and the resilience index | Preconditions for health resilience are: recognition of the global nature of severe health crises and clarity about the roles of actors at all levels of the global health system, egal and policy foundation to guide the response and establish accountability, and the need for a strong and committed health workforce, characterised by health personnel who show up for work that might be difficult and dangerous.  According to this framework, health systems are aware, diverse, self-regulating, integrated and adaptive. | This is one of the strongest frameworks, presented pre-conditions, characteristics and suggested indicators for the resilience index. |
